# Dynamic Displacement Estimation for Long-Span Bridges Using Acceleration and Heuristically Enhanced Displacement Measurements of Real-Time Kinematic Global Navigation System

**DOI:** 10.3390/s20185092

**Published:** 2020-09-07

**Authors:** Kiyoung Kim, Hoon Sohn

**Affiliations:** 1 Research Center for Smart Submerged Floating Tunnel System, Korea Advanced Institute of Science and Technology, Daejeon 34141, Korea; kiyoungkim@kaist.ac.kr; 2 Department of Civil and Environmental Engineering, Korea Advanced Institute of Science and Technology, Daejeon 34141, Korea

**Keywords:** Kalman filter, data fusion, RTK-GNSS, dynamic displacement, bias, modified heuristic drift reduction, accuracy, sampling frequency

## Abstract

In this paper, we propose a dynamic displacement estimation method for large-scale civil infrastructures based on a two-stage Kalman filter and modified heuristic drift reduction method. When measuring displacement at large-scale infrastructures, a non-contact displacement sensor is placed on a limited number of spots such as foundations of the structures, and the sensor must have a very long measurement distance (typically longer than 100 m). RTK-GNSS, therefore, has been widely used in displacement measurement on civil infrastructures. However, RTK-GNSS has a low sampling frequency of 10–20 Hz and often suffers from its low stability due to the number of satellites and the surrounding environment. The proposed method combines data from an RTK-GNSS receiver and an accelerometer to estimate the dynamic displacement of the structure with higher precision and accuracy than those of RTK-GNSS and 100 Hz sampling frequency. In the proposed method, a heuristic drift reduction method estimates displacement with better accuracy employing a low-pass-filtered acceleration measurement by an accelerometer and a displacement measurement by an RTK-GNSS receiver. Then, the displacement estimated by the heuristic drift reduction method, the velocity measured by a single GNSS receiver, and the acceleration measured by the accelerometer are combined in a two-stage Kalman filter to estimate the dynamic displacement. The effectiveness of the proposed dynamic displacement estimation method was validated through three field application tests at Yeongjong Grand Bridge in Korea, San Francisco–Oakland Bay Bridge in California, and Qingfeng Bridge in China. In the field tests, the root-mean-square error of RTK-GNSS displacement measurement reduces by 55–78 percent after applying the proposed method.

## 1. Introduction

It is quite challenging to measure displacement for large-scale civil infrastructure, especially for long-span bridges and high-rise buildings. Unlike acceleration and velocity, measuring displacement requires a fixed reference point, which acts as ground zero so that the displacement of a measurement point on an object of interest can be acquired as a change of distance from the reference point [1]. Contact type displacement sensors such as LVDT (linear variable differential transducer) connect a measurement point and a reference point physically. This connecting work can be very cumbersome and complex, especially for civil structures where the measurement and the reference point are too far to connect the two points with a sensor [2,3]. Noncontact type displacement sensors, such as laser Doppler vibrometer (LDV) and light detection and ranging (LiDAR), remove the shortcomings of the contact type sensors by emitting laser on a surface of a structure and measuring the time-of-flight of the laser [4,5]. However, it is still difficult to find a sensor installation point due to their limited measurement ranges (typically less than 200 m), because the intensity of the reflected laser beam should not be too small compared to the emitted laser beam and the noise in photodetector measurements [1].

Due to the physical limitations of the displacement sensors, RTK-GNSS has been widely used in the civil engineering field [6,7,8,9,10]. RTK-GNSS utilizes two receivers denoted as a rover and a base, which are attached to a measurement and a reference point, respectively. The rover and the base receive the same signals from the same group of satellites; the signals captured by the rover and the base share almost identical error since the transmission paths of the two signals are almost identical [11]. The error, therefore, can be highly reduced (typically from 5–50 m to 1–8 cm) by subtracting the base signal from the rover signal. Since the base and the rover can be installed anywhere unless the antennae of the base and the rover are closed or blocked by an obstacle, RTK-GNSS is almost free from the aforementioned limitation of displacement sensors. However, RTK-GNSS has low sampling rate, which is typically limited to 10 Hz. Also, the displacement measurement accuracy is poorer than other displacement sensors. For example, when the distance between the rover and the base is 1 km, the displacement accuracy is 2–11 mm and 9–22 mm respectively for horizontal and vertical direction [12].

Especially, RTK-GNSS has a high level of low-frequency noise in its displacement measurement. The low-frequency noise leads to a fluctuation of about ±20 mm in the measurement [7]. The low-frequency noise typically comes from 1/f noise (i.e., flicker noise) generated in electric circuits and parts [13], and multipath error of satellite signals [14]. A GNSS signal transmitted from a satellite should arrive at an antenna directly, but also can be reflected on surfaces of various obstacle structures or the ground before reaching to the antenna. The antenna receives the reflected signal right after the direct signal arrives. If the reflected signal is strong enough, the resultant signal has an error called ‘multipath’ [15]. Multipath noise is omnipresent since it is challenging to find a place where no obstacle exists around a measurement point [16].

To overcome the drawbacks of the current sensors, many pieces of research have been conducted on the fusion of measurements from multiple heterogeneous sensors. Traditionally, Kalman filtering has been widely employed to fuse multiple sensors in the field of autonomous vehicles and simultaneous localization and mapping (SLAM) [17,18,19]. Two data fusion schemes are commonly used—loosely-coupled and tightly-coupled Kalman filters [20,21]. It is known that a tightly-coupled Kalman filter has a better performance in vehicle position estimation than loosely-coupled one [22]. In the civil engineering field, Kalman filtering has been mainly used for the estimation of structural displacement by multi-rate data fusion of acceleration and displacement measurements. Smyth and Wu [23] adopted loosely-coupled Kalman filtering without considering acceleration bias, and Kim et al. [24] also proposed a loosely-coupled Kalman filter based on error dynamics. Kim and Sohn [1] introduced a smoothing based Kalman filtering for near-online enhancement of the precision of estimation, and Kim et al. [25] proposed a displacement estimation system based on a force-balanced accelerometer and an RTK-GNSS receiver. However, a high level of low-frequency noise in a RTK-GNSS displacement measurement leads the methods to high estimation error. Since the double integration of the acceleration measurement is corrected by the displacement measurement in Kalman filtering, the noise error cannot be reduced properly by Kalman filter only.

This paper proposed a new dynamic displacement estimation method, which utilizes acceleration measured by an accelerometer and displacement by an RTK-GNSS receiver. The proposed method consists of three parts. First, the low-frequency error in an RTK-GNSS measurement is roughly estimated by modified heuristic drift reduction (MHDR) and the RTK-GNSS measurement is corrected by the estimated low-frequency error. Then the corrected RTK-GNSS measurement is applied to two-stage Kalman filter (TKF) to estimate not only acceleration, velocity, and displacement but also a residual low-frequency error in the corrected RTK-GNSS displacement measurement [26]. The proposed method enhances the accuracy and sampling rate of RTK-GNSS displacement measurement, and estimates the displacement, velocity, and acceleration simultaneously and in real-time.

The remaining part of the paper is organized as follows. Section 2 presents the theoretical description of the proposed method, and then a series of lab-scale tests and field tests and the test results are discussed in Section 3 and Section 4. Finally, concluding remarks are made in Section 5.

## 2. Proposed Dynamic Displacement Estimation Method

The proposed method uses an acceleration measurement from an accelerometer, and a displacement measurement and an error standard deviation estimate from an RTK-GPS sensor at a time step. Also, the proposed method assumes that an accelerometer measures acceleration with higher sampling frequency and an RTK-GNSS sensor measures displacement with lower sampling frequency. Typically, acceleration is measured with 100 Hz of sampling frequency and displacement is measured with 10 Hz for many civil structures.

### 2.1. Schematics of the Proposed Dynamic Displacement Estimation Method

**Figure 1:**
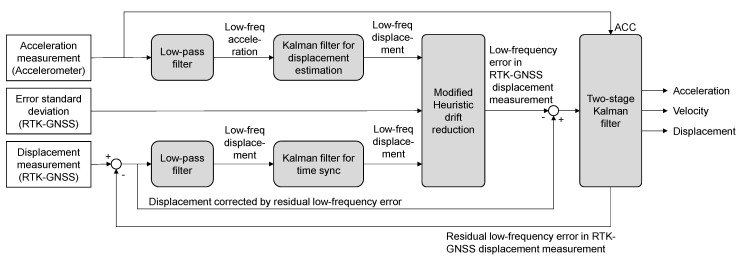
Schematic diagram of the proposed method.

The proposed method is composed of TKF and MHDR as shown in Figure 1. MHDR corrects a low-frequency error in the RTK-GNSS displacement measurement, and TKF fuses the acceleration measurement and the displacement measurement corrected by MHDR for the displacement estimation with better accuracy and 100 Hz sampling rate.

At each time step, MHDR estimates the low-frequency error in the RTK-GNSS displacement measurement by determining the sign (i.e., plus or minus) and the magnitude of the error. First, a low-pass filtered displacement measurement of RTK-GNSS and a lowpass-filtered acceleration measurement are passed to MHDR. The low-pass filtered acceleration and displacement are passed to two conventional Kalman filters as inputs and two displacement estimates are produced as outputs. MHDR compares two displacement estimates and determines the sign of the low-frequency error in the RTK-GNSS displacement measurement, under an assumption that the low-frequency error is generally larger than the error contained in the displacement estimated from the low-pass filtered acceleration. Since a high-fidelity force-balance type accelerometer is typically applied to the health monitoring system of a long-span bridge for the good low-frequency measurement performance, the sign of the low-frequency error can be determined effectively by the assumption. Also, the magnitude of the error is determined based on the error standard deviation of the RTK-GNSS measurement provided by an RTK-GNSS chipset. Then, the raw RTK-GNSS measurement is corrected by subtracting the estimated low-frequency error of RTK-GNSS displacement measurement.

The RTK-GNSS measurement corrected by MHDR and the raw acceleration measurement is passed to TKF, in which the two values are fused to estimate dynamic displacement, velocity, and acceleration with better accuracy and sampling frequency. TKF also estimates low-frequency errors in the RTK-GNSS displacement measurement. At a certain time step, TKF receives the acceleration measurement captured by an accelerometer and the corrected displacement which was measured by an RTK-GNSS receiver and then corrected by MHDR. Here, the corrected displacement measurement still contains low-frequency error as well as the true displacement and a high-frequency noise process. TKF assumes that the low-frequency error is output due to an unknown input of a system, and estimates displacement, velocity, acceleration, and the unknown input and transmits the unknown input to MHDR. MHDR compares the input estimate and the low-frequency component of the acceleration measurement and adjusts the displacement measurement utilizing the difference between the input estimate and the displacement measurement.

### 2.2. Modified Heuristic Drift Reduction for Enhancing RTK-GNSS Displacement Measurement

All RTK-GNSS signal suffers from a high level of low-frequency noise. The low-frequency noise typically comes from 1/f noise (i.e., flicker noise), a kind of colored noise, generated in electric circuits, and multipath of satellite signals. A GNSS signal from a satellite can arrive at an antenna directly, but also can be reflected on surfaces of various obstacle structures and the ground before reaching the antenna. The antenna receives the reflected signal right after the direct signal is arrived. If the reflected signals are strong enough, the resultant signal has an error called multipath. The multipath error is omnipresent since it is challenging to find a place where no obstacle exists around a measurement point.

In this paper, the multipath error is mitigated with MHDR, which is a modified version of heuristic drift reduction (HDR) [27]. HDR is a type of nonlinear signal correction method and was originally developed for removing small low-frequency drift of low-cost gyro sensor, which is widely used in vehicle position tracking. Since the gyro sensor measures angular velocity, it is indispensable to integrate the angular velocity measurement to obtain angular displacement. However, in the process of numerical integration, the low-frequency drift is accumulated and amplified, and the accumulation of the drift leads to a large estimation error. HDR effectively and efficiently removes the low-frequency drift to enhance final estimation accuracy.

However, HDR has a few problems in applying to the mitigation of RTK-GNSS’s low-frequency error. First, RTK-GNSS’s low-frequency error has a relatively large (up to 20 mm) compared to the low-frequency drift of gyros. The threshold, therefore, should be manually set to a high value to identify the low-frequency error, but it leads to the reduction of real vibrations of a structure. Also, the low-frequency error cannot be identified when it is mixed with the structural vibration of civil structures. Note that the structural vibration of large-scale civil structures often has low-frequency vibration under 0.1 Hz due to traffic load and wind load. By utilizing acceleration and displacement measurement, MHDR removes the threshold and automatically estimates the sign and the magnitude of the low-frequency error.

The first step of MHDR is the lowpass filtering of acceleration and displacement measurement and Kalman filtering. Let $\ddot{x}\left(k\right)$, $\dot{x}\left(k\right)$ and x(k) be true acceleration, velocity, and displacement at discrete time steps respectively for k=0, 1, 2, ⋯, and the time increment between two adjacent time steps is Δt, which is assumed to be a constant. Also, let an acceleration measurement ${\ddot{x}}_{m}^{a}\left(k\right)$ by an accelerometer at the time step k consist of true acceleration $\ddot{x}\left(k\right)$, bias $b_{m}^{a}\left(k\right)$ and zero-mean Gaussian white noise $w_{m}^{a}\left(k\right)$

(1) $${\ddot{x}}_{m}^{a}\left(k\right)\;=\ddot{x}\left(k\right)+b_{m}^{a}\left(k\right)+w_{m}^{a}\left(k\right)$$

Since the signs of $b_{a}\left(k\right)$ and $w_{a}\left(k\right)$ have no significant physical meaning, Equation (1) can be rewritten as

(2) $$\ddot{x}\left(k\right)={\ddot{x}}_{m}^{a}\left(k\right)+b_{m}^{a}\left(k\right)+w_{m}^{a}\left(k\right)$$

Similarly, for the displacement measurement of RTK-GNSS sensor, let the displacement measurement $x_{m}^{r}\left(k\right)$ be composed of true displacement x(k), and zero-mean white noise $w_{m}^{r}\left(k\right)$

(3) $$x\left(k\right)=x_{m}^{r}\left(k\right)+w_{m}^{r}\left(k\right)$$

Let lowpass-filtered acceleration and displacement measurements be ${\ddot{x}}_{l}^{a}\left(k\right)$ and $x_{l}^{r}\left(k\right)$. Also, let the lowpass-filtered true displacement and velocity be $x_{l}\left(k\right)$ and ${\dot{x}}_{l}\left(k\right)$, and bias in ${\ddot{x}}_{l}^{a}\left(k\right)$ be $b_{l}^{a}\left(k\right)$. Then the conventional Kalman filter applied to the low-pass filtered acceleration measurement is based on the state-space model in Equations (4) and (5)

(4) $$x_{l}^{a}\left(k+1\right)=A_{al}x_{l}^{a}\left(k\right)+B_{al}{\ddot{x}}_{l}^{a}\left(k\right)+G_{al}w_{l}^{a}\left(k\right)$$

(5) $$y_{al}\left(k\right)=H_{al}x_{l}^{a}\left(k\right)+v_{l}^{a}\left(k\right)$$

where $x_{l}^{a}\left(k\right)={\left(\begin{array}{cc} x_{l}\left(k\right) & b_{l}^{a}\left(k\right) \end{array}\right)}^{T}$, $A_{al}=\left[\begin{array}{cc} 1 & \Delta t\\ 0 & 1 \end{array}\right]$, $B_{al}={\left[\begin{array}{cc} 0.5\Delta t^{2} & \Delta t \end{array}\right]}^{T}$, $G_{al}={\left[\begin{array}{cc} 0.5\Delta t^{2} & \Delta t \end{array}\right]}^{T}$, $H_{al}=\left[\begin{array}{cc} 1 & 0 \end{array}\right]$ and $v_{l}^{a}$ is a zero-mean white noise measurement process. Also, the space-state models of the conventional Kalman filter applied to the low-pass-filtered displacement measurement are

(6) $$x_{l}\left(k+1\right)=x_{l}^{r}\left(k\right)+w_{l}^{r}\left(k\right)$$

(7) $$y_{l}^{r}\left(k\right)=x_{l}\left(k\right)+v_{l}^{r}\left(k\right)$$

where $w_{rl}\left(k\right)$ and $v_{rl}\left(k\right)$ are a zero-mean and measurement white noise processes. Here, $y_{l}^{a}\left(k\right)$ and $y_{l}^{r}\left(k\right)$ are set to zero in every time step, and the error covariances of $w_{l}^{a}\left(k\right)$, $v_{l}^{a}\left(k\right)$, $w_{l}^{a}\left(k\right)$, $v_{l}^{r}\left(k\right)$ are set to the same value. Let ${\hat{x}}_{l}^{a}\left(k\right)={\left(\begin{array}{cc} {\hat{x}}_{l}^{a}\left(k\right) & {\hat{b}}_{l}^{a}\left(k\right) \end{array}\right)}^{T}$, where ${\hat{x}}_{l}^{a}\left(k\right)$ and ${\hat{b}}_{l}^{a}\left(k\right)$ are the displacement and bias estimates from Equations (4) and (5) and ${\hat{x}}_{l}^{r}\left(k\right)$ be the estimates from Equations (6) and (7), then this setup gives rise to the result that the magnitudes of ${\hat{x}}_{l}^{a}\left(k\right)$ and ${\hat{x}}_{l}^{r}\left(k\right)$ reduces by half than the estimates with real displacement measurements since Kalman filter is a weighted average of a prior estimate and a measurement based on the error covariance. However, the purpose of the proposed Kalman filter is just to compare the two estimates ${\hat{x}}_{l}^{a}\left(k\right)$ and ${\hat{x}}_{l}^{r}\left(k\right)$, the incorrectness caused by the setup has no problem in this case.

The second step of MHDR is determining the sign and the magnitude of low-frequency error in the displacement measurement. If ${\ddot{x}}_{m}^{a}\left(k\right)$ and $x_{m}^{r}\left(k\right)$ have no low-frequency error, then ${\hat{x}}_{l}^{a}\left(k\right)$ and ${\hat{x}}_{l}^{r}\left(k\right)$ have the only dynamic response of the structure and should be the same. However, in a real environment, ${\ddot{x}}_{m}^{a}\left(k\right)$ and $x_{m}^{r}\left(k\right)$ have some level of low-frequency errors. Let the low-frequency errors contained in ${\hat{x}}_{l}^{a}\left(k\right)$ and ${\hat{x}}_{l}^{r}\left(k\right)$ be $e_{l}^{a}\left(k\right)$ and $e_{l}^{r}\left(k\right)$, respectively. Then ${\hat{x}}_{l}^{a}\left(k\right)$ and ${\hat{x}}_{l}^{r}\left(k\right)$ can be expressed as

(8) $${\hat{x}}_{l}^{a}\left(k\right)=x\left(k\right)+e_{l}^{a}\left(k\right)$$

(9) $${\hat{x}}_{l}^{r}\left(k\right)=x\left(k\right)+e_{l}^{r}\left(k\right)$$

Subtracting Equation (8) from Equation (9) leads to

(10) $${\hat{x}}_{l}^{r}\left(k\right)-{\hat{x}}_{l}^{a}\left(k\right)=e_{l}^{r}\left(k\right)-e_{l}^{a}\left(k\right)$$

In MHDR, it is assumed that stochastically $E{\left[e_{l}^{r}\left(k\right)\right]}^{2}\geq E{\left[e_{l}^{a}\left(k\right)\right]}^{2}$. Based on the assumption, the sign of $e_{l}^{r}\left(k\right)$ can be determined as follows:

(a) $e_{l}^{r}\left(k\right)-e_{l}^{a}\left(k\right)\geq 0$ for $e_{l}^{r}\left(k\right)\geq 0$ and $e_{l}^{a}\left(k\right)\geq 0$
(b) $e_{l}^{r}\left(k\right)-e_{l}^{a}\left(k\right)<0$ for $e_{l}^{r}\left(k\right)<0$ and $e_{l}^{a}\left(k\right)<0$
(c) $e_{l}^{r}\left(k\right)-e_{l}^{a}\left(k\right)\geq 0$ for $e_{l}^{r}\left(k\right)\geq 0$ and $e_{l}^{a}\left(k\right)<0$
(d) $e_{l}^{r}\left(k\right)-e_{l}^{a}\left(k\right)<0$ for $e_{l}^{r}\left(k\right)<0$ and $e_{l}^{a}\left(k\right)\geq 0$

**Figure 2:**
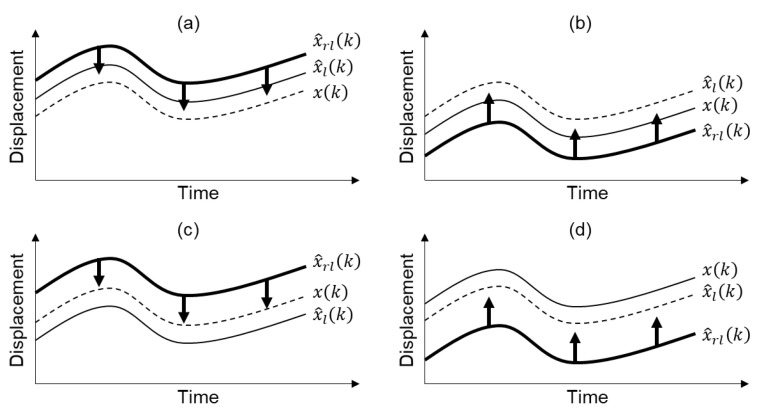
The overall concept of how to determine the sign of $e_{l}^{r}\left(k\right)$, in case of (**a**) $e_{l}^{r}\left(k\right)\geq 0$ and $e_{l}^{a}\left(k\right)\geq 0$, (**b**) $e_{l}^{r}\left(k\right)<0$ and $e_{l}^{a}\left(k\right)<0$, (**c**) $e_{l}^{r}\left(k\right)\geq 0$ and $e_{l}^{a}\left(k\right)<0$, and (**d**) $e_{l}^{r}\left(k\right)<0$ and $e_{l}^{a}\left(k\right)\geq 0$. The thick black arrows indicate the direction of correction.

In cases (a) and (c), the sign of $e_{l}^{r}\left(k\right)$ is estimated as plus and ${\hat{x}}_{l}^{r}\left(k\right)$ would be adjusted to decrease, and in cases (b) and (d), the sign of $e_{l}^{r}\left(k\right)$ is estimated as minus and thus ${\hat{x}}_{l}^{r}\left(k\right)$ would increase. The overall concept of the sign determination of $e_{l}^{r}\left(k\right)$ is illustrated in Figure 2. It is easily concluded from Figure 2 that MHDR decreases ${\hat{x}}_{l}^{r}\left(k\right)$ when ${\hat{x}}_{l}^{r}\left(k\right)\geq {\hat{x}}_{l}^{a}\left(k\right)$ and increases when ${\hat{x}}_{l}^{r}\left(k\right)<{\hat{x}}_{l}^{a}\left(k\right)$.

To determine the magnitude of low-frequency error of the RTK-GNSS displacement, MHDR utilizes error standard deviation value $\sigma_{m}^{r}\left(k\right)$, which is provided by an RTK-GNSS sensor automatically at each time step. Even though there are differences in technical details of the signal processing algorithm, the RTK-GNSS sensor employs extended Kalman filter (EKF) for RTK to estimate pseudo-range, velocity, and single-difference carrier-phase biases using double-difference phase-range and pseudo-range measurements. EKF constructs a measurement error covariance matrix, which is calculated using the variances of the state variables. The RTK-GNSS sensor computes $\sigma_{m}^{r}\left(k\right)$ as a form of the standard deviation of displacement measurement, by combining the variance of each state variable using error propagation principle.

In MHDR, the magnitude of error produced at the time step k is estimated using $\sigma_{m}^{r}\left(k\right)$, the cutoff frequency $f_{c}$ of the lowpass filter and the sampling frequency $f_{n}$ of the displacement measurement of RTK-GNSS. In this procedure, it is assumed that the low-frequency error is a narrow-band process whose peak value is $\sigma_{m}^{r}\left(k\right)$ and frequency is $f_{c}/2$, since $f_{c}$ should be set to a small value. Then the time taken for the low-frequency error to change from 0 to $\sigma_{m}^{r}\left(k\right)$ can be calculated as $1/2f_{c}$, and the increased value of the low-frequency error at each time step can be estimated as

(11) $$i_{c}\left(k\right)=\frac{2\sigma_{m}^{r}\left(k\right)f_{c}}{f_{n}}$$

**Figure 3:**
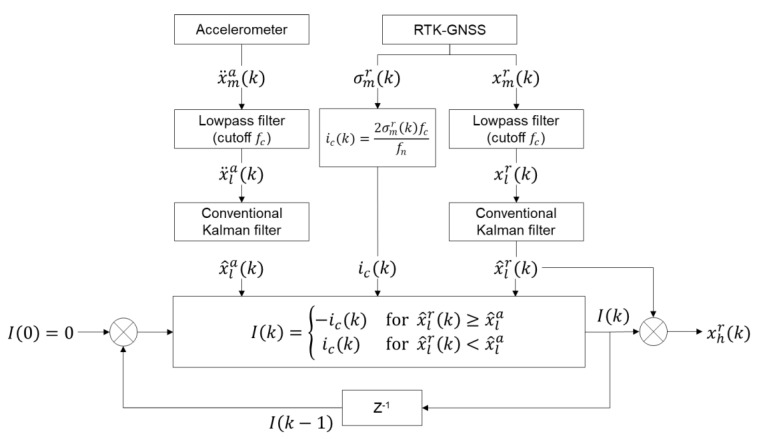
Block diagram of modified heuristic drift reduction. The MHDR mitigates the low-frequency error contained in the RTK-GNSS displacement signal using acceleration.

Note that, in this paper, the values of $f_{c}$ and $f_{n}$ are 0.1 and 10 Hz, respectively, therefore $i_{c}\left(k\right)=\sigma_{m}^{r}\left(k\right)/50$. Then, the total error I(k) at time step k is determined by adding $i_{c}\left(k\right)$ in Equation (11) to I(k), i.e., $I\left(k\right)=I\left(k-1\right)+i_{c}\left(k\right)$, and the corrected RTK-GNSS measurement, denoted by $x_{h}^{r}\left(k\right)$, is calculated by subtracting $i_{c}\left(k\right)$ from $x_{h}^{r}\left(k\right)$. The overall procedure proposed MHDR in Section 2.2 is illustrated in Figure 3.

### 2.3. State-Space Model for Displacement Estimation with Two-Stage Kalman Filter

In Equation (2), let $\delta \ddot{x}\left(k\right)=\ddot{x}\left(k\right)-{\ddot{x}}_{m}^{a}\left(k\right)$, which means the error between true acceleration and measured acceleration. Then, $\delta \ddot{x}\left(k\right)$ has the form of

(12) $$\delta \ddot{x}\left(k\right)=b_{m}^{a}\left(k\right)+w_{m}^{a}\left(k\right)$$

Integrating Equation (12) once, the error propagation model equation can be obtained as

(13) $$\delta \dot{x}\left(k+1\right)=\delta \dot{x}\left(k\right)+b_{m}^{a}\left(k\right)\Delta t+w_{m}^{a}\left(k\right)\Delta t$$

where $\delta \dot{x}\left(k\right)=\dot{x}\left(k\right)-{\dot{x}}_{m}^{a}\left(k\right)$, and ${\dot{x}}_{m}^{a}\left(k\right)$ is the velocity calculated from the acceleration measurement of an accelerometer.

The error propagation model of displacement can be obtained by integrating Equation (13)

(14) $$\delta x\left(k+1\right)=\delta x\left(k\right)+\delta \dot{x}\left(k\right)\Delta t+\frac{1}{2}b_{m}^{a}\left(k\right)\Delta t^{2}+\frac{1}{2}w_{m}^{a}\left(k\right)\Delta t^{2}$$

In the case that Δt is small enough and $b_{m}^{a}\left(k\right)$ is slowly varying along time, $b_{m}^{a}\left(k\right)$ can be treated as a piecewise constant

(15) $$b_{m}^{a}\left(k+1\right)=b_{m}^{a}\left(k\right)$$

For the corrected displacement of RTK-GNSS sensor, adding a bias term $b_{m}^{r}\left(k\right)$ in Equation (3) (i.e., $x_{h}^{r}\left(k\right)=x\left(k\right)+b_{m}^{r}\left(k\right)+w_{m}^{r}\left(k\right)$) and subtracting $x_{m}^{a}\left(k\right)$ on both sides leads to the following equation

(16) $$x_{h}^{r}\left(k\right)-x_{m}^{a}\left(k\right)=\delta x\left(k\right)+b_{m}^{r}\left(k\right)+w_{m}^{r}\left(k\right)$$

A state vector is defined as Equation (17) so that every physical quantity and bias are included as state variables.

(17) $$x_{a}\left(k\right)=\left(\begin{array}{c} \delta x\left(k\right)\\ \delta \dot{x}\left(k\right)\\ b_{m}^{a}\left(k\right)\\ b_{m}^{r}\left(k\right) \end{array}\right)$$

Combining Equations (13), (14) and (17), the transition equation of the state-space model is constructed as

(18) $$x_{a}\left(k+1\right)=A_{a}x_{a}\left(k\right)+G_{a}w_{a}\left(k\right)$$

where

$$A_{a}=\left[\begin{array}{cccc} 1 & \Delta t & 0.5\Delta t^{2} & 0\\ 0 & 1 & \Delta t & 0\\ 0 & 0 & 1 & 0\\ 0 & 0 & 0 & 1 \end{array}\right],\;G_{a}=\left[\begin{array}{ccc} 0.5\Delta t^{2} & 0 & 0\\ \Delta t & 0 & 0\\ 0 & 1 & 0\\ 0 & 0 & 1 \end{array}\right],\;w_{a}\left(k\right)=\left(\begin{array}{c} w_{m}^{a}\left(k\right)\\ w_{b}^{a}\left(k\right)\\ w_{b}^{r}\left(k\right) \end{array}\right)$$

Here, $w_{b}^{a}\left(k\right)$ and $w_{b}^{r}\left(k\right)$ are the process noise of biases $b_{m}^{a}\left(k\right)$ and $b_{m}^{r}\left(k\right)$, respectively. Also, the observation equation of the Kalman filter model is obtained from Equation (16)

(19) $$y\left(k\right)=H_{a}x_{a}\left(k\right)+w_{m}^{r}\left(k\right)$$

where

$$y\left(k\right)=x_{h}^{r}\left(k\right)-x_{m}^{a}\left(k\right),\;H_{a}=\left[\begin{array}{cccc} 1 & 0 & 0 & 1 \end{array}\right]$$

Equation (18) can be divided into two equations as shown in Equatons (20) and (21): one equation whose state vector is composed of physical quantities, and the other equation whose state vector is composed of bias.

(20) $$x\left(k+1\right)=Ax\left(k\right)+Bb\left(k\right)+Gw_{m}^{a}\left(k\right)$$

(21) $$b\left(k+1\right)=b\left(k\right)+w_{b}\left(k\right)$$

where

$$\begin{array}{c} x\left(k\right)=\left(\begin{array}{c} \delta x\left(k\right)\\ \delta \dot{x}\left(k\right) \end{array}\right),\;b\left(k\right)=\left(\begin{array}{c} b_{m}^{a}\left(k\right)\\ b_{m}^{r}\left(k\right) \end{array}\right),\;\\ A=\left[\begin{array}{cc} 1 & \Delta t\\ 0 & 1 \end{array}\right],\;B=\left[\begin{array}{cc} 0.5\Delta t^{2} & 0\\ \Delta t & 0 \end{array}\right],\;G=\left[\begin{array}{c} 0.5\Delta t^{2}\\ \Delta t \end{array}\right],\;w_{b}\left(k\right)=\left(\begin{array}{c} w_{b}^{a}\left(k\right)\\ w_{b}^{r}\left(k\right) \end{array}\right) \end{array}$$

Similarly, Equation (19) can be modified with the state vector used in Equation (20).

(22) $$y=Hx\left(k\right)+Cb\left(k\right)+w_{m}^{r}\left(k\right)$$

where

$$H=\left[\begin{array}{cc} 1 & 0 \end{array}\right],\;C=\left[\begin{array}{cc} 0 & 1 \end{array}\right]$$

### 2.4. Two-Stage Kalman Filter

In TKF [28], acceleration and a corrected displacement measurement by MHDR are fused to produce a displacement estimate with a high sampling rate and no integration error. As mentioned in Section 2, the acceleration measurement has a high sampling rate and precision, but the bias is accumulated through double integration for conversion to displacement. On the other hand, the displacement measurement has a low sampling rate and precision, and its bias can be neglected since no integration is required and bias does not accumulate.

Using the state-space model shown in Equations (20) and (22), x(k) is estimated in TKF. In Stage 1, x(k) is estimated ignoring b(k), and the equations are reduced to the following forms

(23) $$x\left(k+1\right)=Ax\left(k\right)+Gw_{m}^{a}\left(k\right)$$

(24) $$y\left(k\right)=Hx\left(k\right)+w_{m}^{r}\left(k\right)$$

where x(k) is estimated in two steps—prior prediction and posterior correction steps. Define ${\tilde{x}}^{-}\left(k\right)$ and $\tilde{x}\left(k\right)$ are the prior and the posterior estimate of x(k) at time step k, respectively. Then $\tilde{x}\left(k\right)$ is determined through the Kalman filter algorithm described in Equations (25)–(29).

(25) $${\tilde{x}}^{-}\left(k\right)=A\tilde{x}\left(k-1\right)$$

(26) $$P_{x}^{-}\left(k\right)=AP_{x}\left(k-1\right)A^{T}+q\left(k\right)BB^{T}$$

(27) $$K_{x}\left(k\right)=P_{x}^{-}\left(k\right)H^{T}{\left[HP_{x}^{-}\left(k\right)H^{T}+r\left(k\right)\right]}^{-1}$$

(28) $$\tilde{x}\left(k\right)=\left[I-K_{x}\left(k\right)H\right]{\tilde{x}}^{-}\left(k\right)+K_{x}\left(k\right)y\left(k\right)$$

(29) $$P_{x}\left(k\right)=P_{x}^{-}\left(k\right)-K_{x}\left(k\right)HP_{x}^{-}\left(k\right)$$

where $q\left(k\right)=E\left[w_{m}^{a}{\left(k\right)}^{2}\right]$ and $r\left(k\right)=E\left[w_{m}^{r}{\left(k\right)}^{2}\right]$. Note that q(k) is identical to the variance of $w_{m}^{a}\left(k\right)$ because $w_{m}^{a}\left(k\right)$ is a zero-mean process. Therefore, q(k) is treated as a constant under the assumption of wide-sense stationarity.

In Stage 2, $\hat{x}\left(k\right)$ and $\tilde{x}\left(k\right)$ are assumed to be related to each other as

(30) $${\hat{x}}^{-}\left(k\right)={\tilde{x}}^{-}\left(k\right)+U\left(k\right)b\left(k\right)$$

(31) $$\hat{x}\left(k\right)=\tilde{x}\left(k\right)+V\left(k\right)b\left(k\right)$$

where U(k) and V(k) are prior and posterior sensitivity matrices. Note that the values of the matrices are unknown, and they need to be estimated at time step k. U(k) and V(k) can be estimated through a recursion process utilizing $K_{x}\left(k\right)$ calculated in Stage 1 [26]

(32) $$U\left(k\right)=AV\left(k-1\right)-AV\left(k-1\right)Q_{b}\left(k-1\right)P_{b}^{-}{\left(k-1\right)}^{-1}$$

(33) $$V\left(k\right)=U\left(k\right)-K_{x}\left(k\right)S\left(k\right)$$

where S(k)=HU(k)+C and $Q_{b}\left(k\right)$ is an error covariance of b(k).

After U(k) and V(k) are calculated, b(k) can be estimated by following recursion process

(34) $${\hat{b}}^{-}\left(k\right)=\hat{b}\left(k-1\right)$$

(35) $$P_{b}^{-}\left(k\right)=P_{b}\left(k-1\right)+Q_{b}\left(k-1\right)$$

(36) $$K_{b}\left(k\right)=P_{b}^{-}\left(k\right)S^{T}\left(k\right){\left[HP_{x}^{-}\left(k\right)H^{T}+HU\left(k\right)P_{b}^{-}\left(k\right)U\left(k\right)H^{T}+R\left(k\right)\right]}^{-1}$$

(37) $$\hat{b}\left(k\right)=\left(1-K_{b}\left(k\right)S\left(k\right)\right){\hat{b}}^{-}\left(k\right)+K_{b}\left(k\right)r\left(k\right)$$

(38) $$P_{b}\left(k\right)=P_{b}^{-}\left(k\right)-P_{b}^{-}\left(k\right)K_{b}\left(k\right)HU\left(k\right)$$

where ${\hat{b}}^{-}\left(k\right)$ and $\hat{b}\left(k\right)$ are the prior and posterior estimates of b(k), and $P_{b}^{-}\left(k\right)$ and $P_{b}\left(k\right)$ are the error covariance matrices of the prior and posterior estimation, respectively. Also, $K_{b}\left(k\right)$ is the Kalman gain of the bias estimation.

Finally, $\hat{x}\left(k\right)$ is obtained by replacing b(k) in Equation with $\hat{b}\left(k\right)$ obtained from Equation (31)

(39) $$\hat{x}\left(k\right)=\tilde{x}\left(k\right)+V\left(k\right)\hat{b}\left(k\right)$$

and the unknown input u(k) to the system is expressed as

(40) $$u\left(k\right)=\left(U\left(k\right)-AV\left(k-1\right)\right)\hat{b}\left(k\right)$$

Note that the state-space models proposed in the previous studies do not have an observation equation when the displacement is not measured, and a two-stage Kalman estimator can be applied only when intermittent displacement data are measured. However, using the proposed state-space model in this study, V(k) and $\hat{b}\left(k\right)$ in Equation (39) can be updated continuously, and this is a major advantage of the proposed method in terms of accuracy enhancement.

## 3. Lab-Scale Experiment

**Figure 4:**
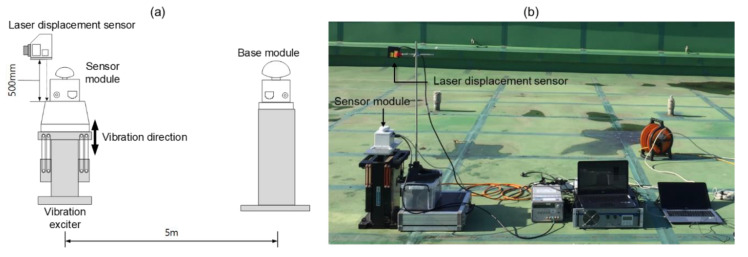
Overview of the lab-scale experiment: (**a**) schematic diagram of experiment configuration and (**b**) a photo taken after the sensor module installation.

A lab-scale experiment was performed to validate the performance of the proposed method. An overview of the test configuration is shown in Figure 4. In this experiment, a set of sensor systems developed by authors and Poongsan FNS Co. [29] was adopted. The sensor system consists of a sensor module, a combination of a triaxial accelerometer, an RTK-GNSS rover chipset and an antenna, and a base module which has an RTK-GNSS base chipset and an antenna. Swift Navigation’s Piksi Multi GNSS chipsets were adopted for the sensor and the base module. The GNSS chipset is low-cost as much as $2300, including two survey grade antennae, but the chipset supports GPS L1/L2, GLONASS G1/G2, BeiDou B1/B2, Galileo E1/E5b, and SBAS constellation. Its elevation mask was set to 10° and sampling frequency was 10 Hz.

The overall setup for the vibration tests is shown in Figure 4. The sensor module is placed and fixed on an APS ElectroSeis vibration exciter for vertical vibration. The base module is installed 5 m away from the sensor module. The reference displacement is measured using a KL-W400 laser displacement sensor, which has 10 μm resolution and ±0.08% linearity. The sensor module measured acceleration and displacement and transmitted the measurements to a computer through a switching hub. A range of 12 to 14 satellites were continuously observed during the experiment.

**Figure 5:**
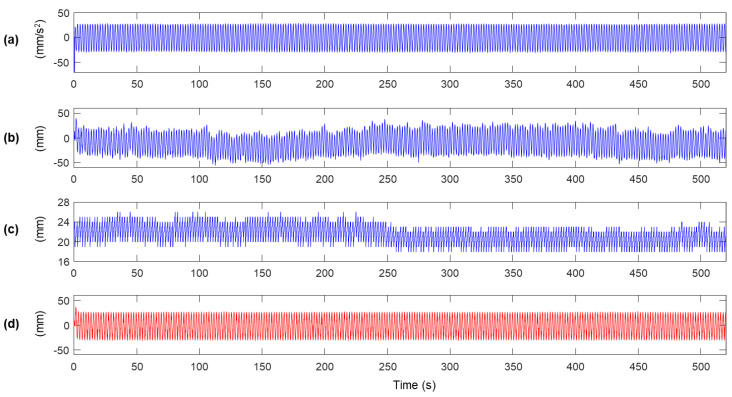
Measurements obtained from the lab-scale experiment: (**a**) acceleration from the accelerometer (${\ddot{x}}_{m}^{a}\left(k\right)$), (**b**) displacement from RTK-GNSS ($x_{m}^{r}\left(k\right)$), (**c**) error standard deviation from RTK-GNSS ($\sigma_{m}^{r}\left(k\right)$), and (**d**) reference displacement from a laser displacement sensor (x(k)).

**Figure 6:**
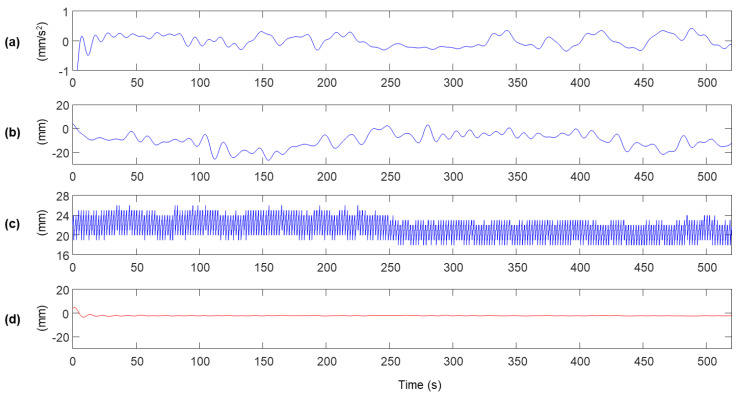
Measurements filtered out with a lowpass filter with 0.1 Hz cutoff frequency: (**a**) ${\ddot{x}}_{l}^{a}\left(k\right)$, (**b**) $x_{l}^{r}\left(k\right)$, (**c**) $\sigma_{m}^{r}\left(k\right)$, and (**d**) $x_{l}\left(k\right)$ Figure 7 compares ${\hat{x}}_{l}^{a}\left(k\right)$ and ${\hat{x}}_{l}^{r}\left(k\right)$, displacements estimated through two conventional Kalman filters described in Equations (4) and (5) and Equations (6) and (7), respectively. Note that the magnitude of ${\hat{x}}_{l}^{r}\left(k\right)$ shown in Figure 7a is exactly half of the magnitude of $x_{l}^{r}\left(k\right)$ (see Figure 6b) due to the zero-measurement applied to the conventional Kalman filter. As mentioned in Section 2.2, the proposed MHDR compares ${\hat{x}}_{l}^{a}\left(k\right)$ and ${\hat{x}}_{l}^{r}\left(k\right)$ to determine the sign of $e_{l}^{r}\left(k\right)$. In Figure 7a, it is clearly shown that the absolute of ${\hat{x}}_{l}^{r}\left(k\right)$ is larger than that of ${\hat{x}}_{l}^{a}\left(k\right)$ at most time steps. Comparing the sign of ${\hat{x}}_{l}^{r}\left(k\right)-{\hat{x}}_{l}^{a}\left(k\right)$ and the $e_{l}^{r}\left(k\right)$ calculated by the sign of ${\hat{x}}_{l}^{r}\left(k\right)-x_{l}\left(k\right)$, the two values are identical in 93.03% of time steps (Figure 7b).

In the experiment, the sensor module was excited vertically with a vibration frequency of 0.5 Hz for 520 s to reproduce the vibration of a bridge span, and only vertical acceleration and displacement were measured. The measurement from the accelerometer and RTK-GNSS built in the sensor module and the reference displacement are shown in Figure 5. The maximum peak of the vibration in the reference shown in Figure 5d is approximately ±25 mm, but the peaks of the displacement measurement of RTK-GNSS are distributed within ±50 mm, as shown in Figure 5b. Also, the error standard deviation of the displacement measurement is distributed from 18 to 26 mm. It can be easily observed that low-frequency noise is relatively large when high error standard deviation is calculated (0–200 s), but the magnitude of the low-frequency noise decreased at 300–450 s, in which the error standard deviation has relatively low values. The characteristics of the low-frequency noise can be identified when the measurement signals are lowpass filtered with $f_{c}=0.1$ Hz cutoff frequency, as shown in Figure 6.

**Figure 8:**
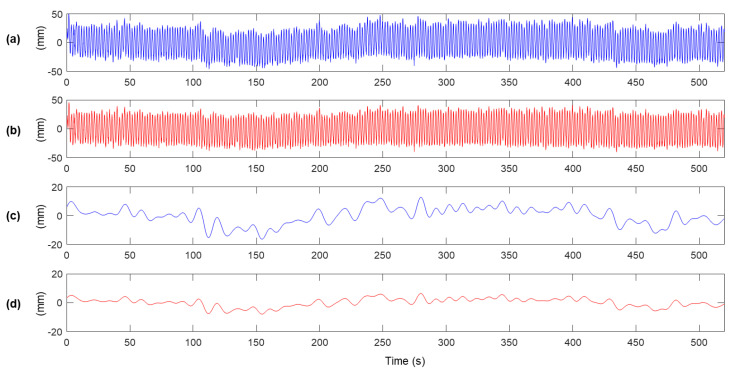
Comparison of low-frequency error before and after MHDR is applied: (**a**) $x_{m}^{r}\left(k\right)$, (**b**) $x_{h}^{r}\left(k\right)$, (**c**) $x_{l}^{r}\left(k\right)$, and (**d**) $x_{lh}^{r}\left(k\right)$.

The measurement displacements before and after MHDR are compared in Figure 8. Figure 8a,b shows $x_{m}^{r}\left(k\right)$ and $x_{l}^{r}\left(k\right)$. The low-frequency components in $x_{m}^{r}\left(k\right)$ is highly reduced after executing MHDR, and Figure 8c,d are the low-frequency components of $x_{m}^{r}\left(k\right)$ and $x_{l}^{r}\left(k\right)$ respectively, produced by a lowpass filter with the cutoff frequency of 0.1 Hz. The low-frequency error observed in Figure 8a,c are highly reduced after applying MHDR as in Figure 8b,d. The RMSEs of the low-frequency components in Figure 8b,d with respect to $x_{l}\left(k\right)$ is 6.48 mm and 3.19 mm. As for the total error in $x_{m}^{r}\left(k\right)$ and $x_{h}^{r}\left(k\right)$ with respect to x(k) are calculated as 8.56 mm and 6.47 mm, respectively. Note that the error in the high-frequency band over 0.1 Hz is calculated as 5.62 mm by the propagation of uncertainty, assuming that the low and the high-frequency band error do not correlate.

**Figure 9:**
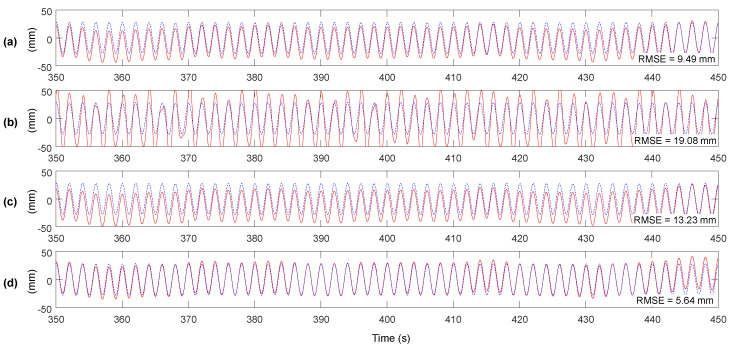
Displacement estimation results and RMSE errors for the method of (**a**) Smyth and Wu [23], (**b**) tightly-coupled and (**c**) loosely-coupled Kalman filter [22], and (**d**) the proposed method.

Figure 9 shows the improvement of accuracy after applying the proposed method, by comparing to the methods proposed by Smyth and Wu [23], and loosely and tightly-coupled Kalman filter [22]. Considering the RMSE of RTK-GNSS displacement measurement is 8.56 mm, the RMSE of the proposed method including MHDR is 5.64 mm, representing a 33.6% reduction. However, as shown in Figure 9a–c, other methods do not reduce the error of RTK-GNSS displacement considerably. The tightly-coupled and loosely-coupled Kalman filters uses the RTK-GNSS displacement measurement and prior estimate of the Kalman filters as inputs of a weighted average as shown in Equation (28), hence the error in the RTK-GNSS displacement measurement cannot be reduced properly and remains in the final estimate. It implies that the accuracy of RTK-GNSS displacement measurement should be enhanced before applied to a Kalman filter. Note that the parameters of the Kalman filters, q(k) and r(k) in Equations (26) and (27) were set to 1 and 24.47, respectively. The values were determined by a preliminary lab-scale test, in which the acceleromenter and RTK-GNSS module were placed on a fixed ground and variances of noises from the two measurements were calculated.

## 4. Field Tests

**Figure 10:**
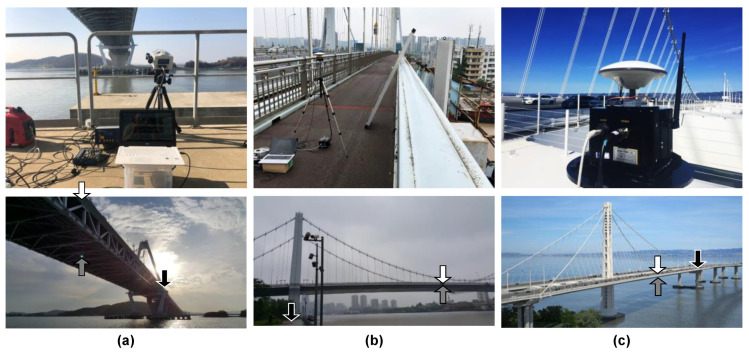
Field tests performed for the validation of the performance of the proposed method at (**a**) Yeongjong Grand bridge, (**b**) Qingfeng bridge, and (**c**) San Francisco–Oakland Bay bridge. The white, black, grey arrows in the bottom pictures represent the locations of sensor modules and base modules, and target points of reference measurements, respectively.

The accuracy of the proposed method was explored through field tests at three long-span bridges—Yeongjong Grand bridge in Korea, Qingfeng bridge in China, and the San Francisco–Oakland bay bridge in California, as shown in Figure 10. In the field tests, the vertical motion of the main span of the bridges was measured by RTK-GNSS and an accelerometer. Yeongjong Grand bridge, a three-dimensional self-anchored suspension bridge, is a part of Incheon International Airport Expressway. The bridge has a main span of 300 m, and the length of the side spans is 120 m. The deck of the bridge consists of two steel truss decks. The upper deck is for vehicles only, but there are two railroads in the middle of the bottom deck for high-speed trains. Qingfeng bridge, located in the city of Ningbo in China, is a suspension bridge with steel box girders, and its longest span length is 280 m. San Francisco–Oakland Bay bridge connects the city of San Francisco and Oakland, and is divided into the east and the west span. The field test was conducted on the east span, which is a three-dimensional self-anchored suspension bridge, and whose main span length is 430 m. The main girder of the bridge is a double-deck type and made of steel box structures.

In the field test at Yeongjong Grand bridge, a Leica GS18T base module was installed on the top of an office building, about 7.7 km away from Leica GS18T rover modules. The rovers are installed on the top of rigid steel columns to minimize multipath error. An accelerometer, Kinemetrics Episensor ES-T, was mounted on the main girder of a truss structure. In the field tests at Qingfeng and San Francisco–Oakland Bay bridge, a sensor system developed by authors and Poongsan FNS Co. were installed. The sensor module was installed on a measurement point, 150 m away from a pylon, and the base module was placed on a nearby pedestrian trail in the Qingfeng bridge test. In the San Francisco–Oakland Bay bridge test, the sensor module was mounted a measurement point 60 m away from the pylon of the bridge, and the base module was installed on the nearest pier, 320 m away from the sensor module.

**Figure 11:**
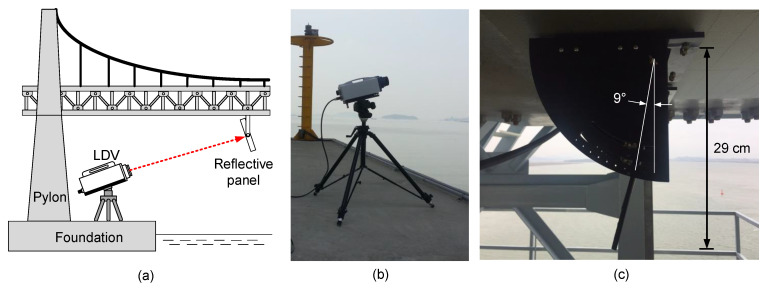
Test setup for the measurement of reference displacement at Yeongjong Grand bridge: (**a**) overall setup diagram of LDV and reflective panel, (**b**) LDV, and (**c**) reflective panel.

To measure a reference displacement of the three bridges, a Polytec RSV-150 laser Doppler vibrometer (LDV) was placed near the foundation of a pylon, as shown in Figure 11a,b. The LDV emits a class 2 laser to secure a long-range out-of-plane movement measurement up to 300 m. However, the measurement quality of the LDV is highly dependent on the light intensity of the returned laser beam. When the surface of a target point is rough and causes a diffuse reflection, or the incidence angle between the surface and the beam is too small, the measurement accuracy of the LDV can be highly degraded and unreliable. The incident angle should be 90° for the best measurement accuracy, but the incident angles of the LDV’s laser beam were limited to 9° at Yeongjong Grand bridge (Figure 11c), 14° for Qingfeng bridge, and 26° for San Francisco–Oakland Bay bridge. To arrange for a surface that the LDV can emit the laser beam with the incident angle of 90°, a reflective panel was installed on the bottom of the girder as shown in Figure 11c. A retroreflective sheet was pasted on the panel to enhance the reflectivity and make sure of regular reflection. The angle of the panel was adjusted so that the incident angle was close to 90°. The measured out-of-plane movement of the panel can be converted to the vertical displacement of the measurement point using simple trigonometry [29].

**Figure 12:**
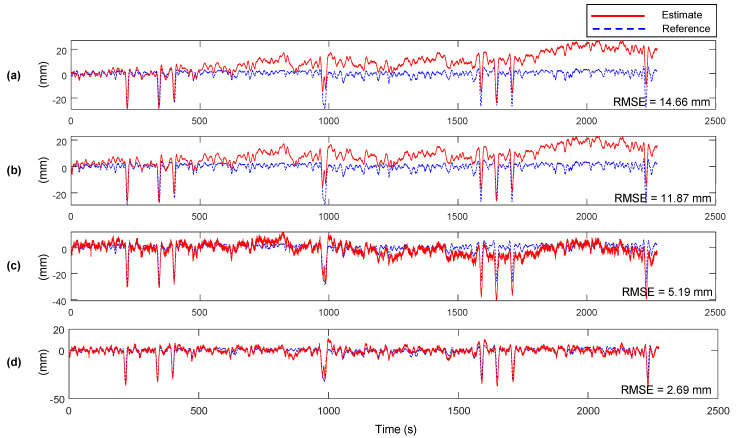
Displacement estimation results and RMSE errors for the Yeongjong Grand bridge: (**a**) the method proposed by Smyth and Wu [23], (**b**) tightly-coupled and (**c**) loosely-coupled Kalman filter [22], and (**d**) the proposed method.

The estimation results, reference displacement measurements at Yeongjong Grand bridge, and RMSE of between the estimates and the references are shown in Figure 12. Note that the values of Kalman filter parameters are set to the same to the lab-scale test in Section 3. The estimation accuracy of the proposed method is compared to the ones of the methods proposed by Smyth and Wu [23], and loosely and tightly-coupled Kalman filter [22]. Whereas the three previous methods cannot reduce the low-frequency error, inherited from the one of RTK-GNSS displacement measurement. The results clearly show that the previous Kalman filtering based displacement estimation methods have a limitation on the estimation accuracy in the presence of low-frequency error. However, the proposed method effectively mitigates the low-frequency error by MHDR and the two-stage Kalman filter.

The estimation results and their RMSEs with respect to the reference measurement in the three field tests are summarized in Table 1. The RMSEs of RTK-GNSS displacement measurements are highly enhanced by MHDR, which corrects low-frequency errors in the RTK-GNSS displacement measurement. Then, two-stage Kalman filter produces the final displacement estimates by correcting high-frequency error and residual low-frequency error. Table 2 compares the performance of the proposed method to the previous Kalman filter based displacement estimation method proposed by Smyth and Wu [23] and loosely and tightly-coupled Kalman filter [22], and the proposed method. Since the proposed method, unlike other previous methods, mitigates the errors with both MHDR and Kalman filtering, the accuracy of the estimates is highly enhanced up to 2.69 mm.

## 5. Conclusions

The study explores a dynamic displacement estimation method based on MHDR and the two-stage Kalman filter, especially for large-scale civil infrastructures. The proposed method fuses the displacement measurement from an RTK-GNSS and the acceleration measurement from an accelerometer. MHDR mitigates low-frequency multipath error, which contaminates RTK-GNSS displacement measurement, up to 74%. Two-stage Kalman filter utilizes the displacement from MHDR and an acceleration measurement for estimating displacement with high accuracy and a high sampling rate. A field test at Yeongjong Grand Bridge shows that the proposed method enhances the RMSE of RTK-GNSS from 5.97 mm to 2.69 mm. However, since both RTK-GNSS and the accelerometer do not have good accuracy when measuring low-frequency vibration under 0.1 Hz, the proposed method also has a limitation in applying to a structure that vibrates at ultra low-frequency or pseudo-statically. To resolve the problems, the authors are planning to introduce vision-based sensing technology in the proposed method. It is well known that vision-based sensors show strong performance in low-frequency vibration measurement, the authors expect that an accelerometer and a vision-based sensor may compensate the drawback effectively.

## Figures and Tables

**Figure 7:**
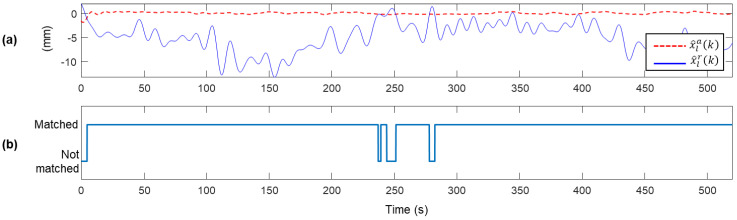
(**a**) Comparison of ${\hat{x}}_{l}^{r}\left(k\right)$ and ${\hat{x}}_{l}^{a}\left(k\right)$ and (**b**) a plot indicating whether the signs of ${\hat{x}}_{l}^{r}\left(k\right)-{\hat{x}}_{l}^{a}\left(k\right)$ and ${\hat{x}}_{l}^{r}\left(k\right)-x_{l}\left(k\right)$ match or not match. The two signs match in 96.63% of the time steps.

**Table 1:** RMSEs of estimates from the measurements of each field test.

(mm)	Yeongjong Grand Bridge	Qingfeng Bridge	San Francisco–Oakland Bay Bridge
RTK-GNSS displacement measurement, $x_{m}^{r}\left(k\right)$	5.97	20.16	13.39
RTK-GNSS displacement after MHDR applied, $x_{h}^{r}\left(k\right)$	3.27	5.49	5.76
Displacement estimate of two-stage Kalman filter, $\hat{x}\left(k\right)$	2.69	5.08	5.44

**Table 2:** Estimation accuracy comparison of the proposed method to the displacement estimation method proposed by Smyth and Wu and loosely and tightly-coupled Kalman filter.

(mm)	Yeongjong Grand Bridge	Qingfeng Bridge	San Francisco–Oakland Bay Bridge
Proposed	2.69	5.08	5.44
Smyth and Wu	14.66	22.13	14.96
Loosely-coupled Kalman filter	11.87	19.90	12.75
Tightly-coupled Kalman filter	5.19	22.89	13.68
